# Evaluation of Incisor Position in a Sample of Orthodontic Patients

**DOI:** 10.3390/diagnostics14182062

**Published:** 2024-09-18

**Authors:** Roberto Rongo, Margherita Maria Eleonora Importuna, Ada Carolina Pango Madariaga, Rosaria Bucci, Vincenzo D’Antò, Rosa Valletta

**Affiliations:** School of Orthodontics, Department of Neurosciences, Reproductive Sciences and Oral Sciences, University of Naples Federico II, Via Pansini, 5, 80131 Naples, Italy; roberto.rongo@unina.it (R.R.); apangom@gmail.com (A.C.P.M.); rosaria.bucci@unina.it (R.B.); valletta@unina.it (R.V.)

**Keywords:** upper and lower incisor position, LIP, UIP, cephalometric analysis, intermaxillary discrepancies, facial divergence, skeletal malocclusion, lip incompetence

## Abstract

Background: To achieve a successful result, the orthodontist must use a systematic approach to plan the orthodontic treatment. Defining the correct position of the upper and lower incisors and evaluating their relationship with intermaxillary discrepancy and facial divergence have been recognized as the starting point for the diagnostic decision regarding extractions and anchorage requirements. The aim of our study was to analyze the relationship between intermaxillary discrepancy (ANPg^), mandibular inclination (SN^GoGn), lip incompetence, and the positioning of the upper and lower incisors (UIPs and LIPs) in a group of orthodontic patients. This retrospective study included 290 lateral cephalograms in 122 males (42.1%) and 168 females (57.9%) aged 8 to 53 years (median 14; interquartile range IQR 12–17). Data were analyzed by means of one-way Analysis of Variance (ANOVA) and linear regression analysis. Results: This study showed a statistically significant increase in LIP values in patients with lip incompetence (*p* < 0.001). Moreover, the distribution of LIPs in groups with various SN^GoGn and ANPg^ angles was significantly different (*p* < 0.001). The regression analysis also showed a positive association between the LIP and SN^GoGn and between the LIP and ANPg^. Conclusion: The LIP presented a statistically significant difference in patients with and without lip incompetence, which varied significantly in subjects with different sagittal malocclusions and vertical face patterns.

## 1. Introduction

A successful orthodontic therapy should be based on a proper diagnosis and a well-defined treatment plan in order to achieve the desired outcome [1,2]. Precisely, in this perspective, a careful assessment of the patient’s face, profile, and dental and skeletal morphological features with the help of cephalometry must be considered during the treatment planning process [3,4,5,6,7]. Defining the ideal position of the upper and lower incisors relative to one other and their supporting bones is one of the main goals of the orthodontic treatment, as the position of these teeth gives harmony and balance to the facial profile and is predictive for post-treatment stability [8,9].

In orthodontic history, researchers such as Downs [10], Steiner [11], McNamara [12], and Arnett et al. [13] have all recommended cephalometric methods for determining the most suitable and stable incisor sagittal position. In 1943, Margolis introduced the Incisor–Mandibular Plane Angle (IMPA) as a metric for evaluating the position of the lower incisor relative to the mandibular plane [14]. However, Tweed identified a significant limitation in this measurement, supporting its susceptibility to mandibular morphology. To address this, Tweed proposed a diagnostic triangle including the FMIA, FMA, and IMPA to better account for vertical skeletal discrepancies in assessing lower incisor position [15,16]. Ricketts further advanced the assessment by introducing an angular cephalometric parameter that measures the angle between the lower incisor axis and the Frankfurt plane (B1 to FH), where the Frankfurt plane remains constant, thus making the measurement dependent solely on B1 inclination [17]. Despite the widespread application of the IMPA, it is known to have limitations, particularly in patients with pronounced vertical dimensions or significant sagittal discrepancies [18]. To address these limitations, two cephalometric values, the lower incisor position (LIP) and the upper incisor position (UIP), were introduced to evaluate the relationship between the anterior dentition limit and vertical and sagittal skeletal patterns.

The LIP measures the distance between the Pogonion (Pg) and a line perpendicular to the Frankfurt plane (Pr-Or) passing through the margin of the lower incisor. This value helps to identify the position of the anterior limit of the dentition [19], consisting of the buccal and lingual cortical plates of the mandibular symphysis, which define the limits of orthodontic tooth movements. Moving an incisor excessively beyond this limit may lead to iatrogenic periodontal issues, such as gingival recession and bone loss, especially in patients with poor oral hygiene or a thin cortical bone [20]. In this perspective, a backward position of the lower incisor in the mandibular symphysis allows the orthodontist to procline the lower incisors’ recovering space in case of crowding. The position of the mandibular incisor is strictly related to lower face aesthetics and long-term occlusion stability [18]. Previous studies showed that different sagittal and vertical discrepancies were associated with different lower incisor positions and inclinations [21,22]. Most cephalometric analyses rely on the angle between the lower incisor axis and the mandibular plane to evaluate the position of the lower incisor. This can be a hindrance to the diagnostic process since the mandibular plane is strongly influenced by facial divergence and the mandibular growth pattern of the patient. Considering this, the usefulness of the LIP as a cephalometric value is easily explained; by only taking into consideration the position of the lower incisor with respect to the mandibular symphysis, the LIP can be a valid helping tool in achieving a comprehensive view of the patient’s condition, leading the clinician to develop a more accurate and customized treatment plan.

While much research has focused on the lower incisors in orthodontics, the maxillary incisors have been less studied. Riedel, in 1952, emphasized the importance of evaluating the axial inclination of the upper incisors, finding a mean angle of 103.5 degrees relative to the SN plane and 111 degrees to the Frankfurt horizontal (FH) in adults and 110 degrees in children [23].

In this study, the upper incisor position (UIP) value has been used to assess the position of the maxillary incisor. The UIP represents the distance between the anterior nasal spine (ANS) and a line perpendicular to the Frankfurt plane (Pr-Or) passing through the margin of the upper incisor. As with the LIP, employing the UIP value in the cephalometric analysis allows us to evaluate the position of the upper incisor without being influenced by either the palatal plane or other landmarks, which could mask the actual protrusion or retrusion of the incisor.

It is also interesting to note how sagittal discrepancies and anterior face height are correlated to lip incompetence, a condition defined by Proffit as lips that are more than 3–4 mm apart and unable to close adequately at rest [24]. This condition is frequently associated with protrusive dentition; in particular, the upper and lower incisors tend to be overly proclined. As a result, many patients seek orthodontic care to decrease this protrusion [25]. However, clinicians must distinguish between incompetent lips, which represent a pathological problem, and prominent lips with a proper seal, which are commonly found in certain ethnic groups. Hassan et al. found that lip incompetence is a multifactorial problem and is frequently associated with sagittal and vertical skeletal discrepancies and is not exclusively caused by bimaxillary dentoalveolar protrusion [26].

A systematic evaluation of lower and upper incisors’ positions in comparison with sagittal discrepancies and vertical skeletal patterns may be a helpful tool to improve the accuracy of treatment planning [27]. 

The aim of our study was to analyze the values of the LIP and UIP in patients with different skeletal (sagittal and vertical) types of malocclusions, with or without lip incompetence. 

## 2. Materials and Methods

### 2.1. Subjects

This research protocol was approved by the Ethics Committee of the University of Naples “Federico II” (121/19; 18 March 2019). 

For this retrospective study, a consecutive sampling of cephalometric radiographs of 290 patients (122 males and 168 females, aged 15.6 ± 6.0 years) were selected from the patients’ database at the Department of Orthodontics of the University of Naples “Federico II” (Table 1). The patients started the orthodontic treatment between January 2018 and December 2019. 

Due to the retrospective design of this study, it was not possible to obtain informed consent from all participants; however, prior to treatment, all patients provided authorization to use their medical records for research purposes.

The teleradiographs were selected based on the following inclusion criteria:

- Caucasian race;
- Age ≥ 8 years, with upper and lower central incisors totally erupted;
- Teleradiographs in the lateral projection that had good quality.

The following conditions were considered exclusion criteria:

- Patients with systemic diseases;
- Patients with genetic syndromes;
- Previous orthodontic treatment and a history of obstructed nose breathing.

### 2.2. Sample Size Calculation

Sample size calculation was performed, considering the differences of the LIP among three groups as the main outcome, and ANOVA was used as a statistical test. Hence, it was found that a sample size of 252 achieved 95% power to detect a medium effect size (f^2^) of 0.025, which was attributable to one independent variable(s) using an F-test with a significance level (alpha) of 0.050. 

### 2.3. Cephalometric Analysis and Clinical Examination

All lateral radiographs were taken in the natural head position before the orthodontic treatment [28,29]. One operator traced all lateral cephalograms with cephalometric software (Delta-Dent, Outside Format, Piolla, Italy).

**Figure 1:**
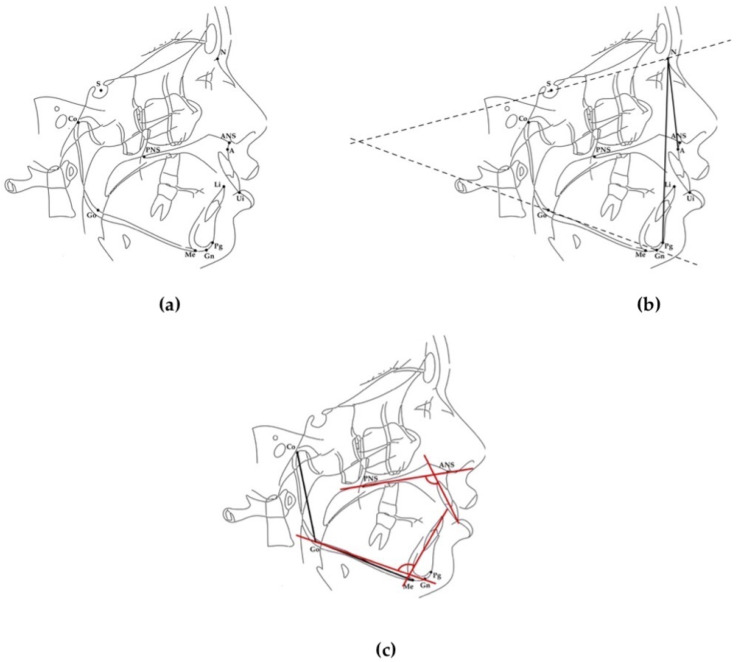
Skeletal landmarks, lines, and measurements used for cephalometric analysis. (**a**) Landmarks: Sellion (S), midpoint of the cavity of sella turcica (hypophyseal fossa); Nasion (N), most anterior point of the junction of the nasal and frontal bone (fronto-nasal suture); Subspinale (A), most posterior point of the frontal concavity of the maxilla between the anterior nasal spine and the most inferior point on the alveolar bone overlying the maxillary incisors; Gonion (Go), midpoint of the curvature at the angle of the mandible; Condylion (Co), highest and most posterior point of the outline of the mandibular condyle; Pogonion (Pg), most anterior point on the contour of the mandibular symphysis; Gnathion (Gn), most anterior inferior point on the curvature of the symphysis of the mandible, halfway between Pg and Me; Menton (Me), most inferior point on the mandibular symphysis; upper incisor (Ui), point on the incisal margin of the upper incisor; lower incisor (Li), point on the incisal margin of the lower incisor; anterior nasal spine (ANS), the anterior tip of the sharp bony process of the maxilla at the lower margin of the anterior nasal opening; posterior nasal spine (PNS), posterior spine of the palatine bone constituting the hard palate. (**b**) Plane and angular measurements: Sellion–Nasion plane (SN), plane formed by connecting S point to N point; mandibular plane (GoGn), plane formed by a line connecting Gonion and Gnathion; Nasion–Point A line (NA), a straight line passing through Nasion and Point A; Nasion–Pogonion line (NPg), a straight line passing through Nasion and Pogonion; SN^GoGn, angle between the Sella–Nasion plane and the mandibular plane; ANPg^, angle between the Nasion–Point A line and the Nasion–Pogonion line. (**c**) Plane and angular measurements: Condylar axis (CoGo), plane formed by a line connecting Condylion and Gonion; mandibular base (GoMe), plane formed by a line connecting Gonion and Menton; mandibular plane (Go-Gn), plane formed by a line connecting Gonion and Gnathion; palatal plane (ANS-PNS), plane formed by a line connecting ANS to PNS; CoGoMe^, angle between condylar axis and mandibular base; ANS-PNS^Ui, angle between long axis of maxillary central incisor and palatal plane; Go-Gn^Li, angle between long axis of mandibular central incisor and mandibular plane.

**Figure 2:**
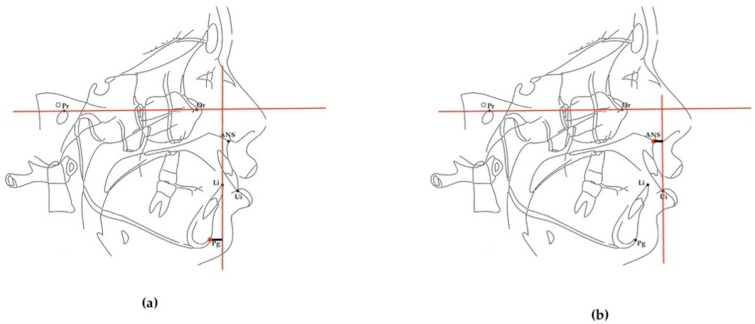
Lower incisor position (LIP) and upper incisor position UIP: (**a**) lower incisor position (LIP), the distance between the perpendicular to the Frankfurt horizontal plane passing through the incisal margin of the mandibular central incisor and Pogonion (Pg); (**b**): upper incisor position (UIP), the distance between the perpendicular to the Frankfurt plane passing through the incisal margin of the maxillary central incisor and the anterior nasal spine (ANS).

Two linear and five angular cephalometric measurements were used to assess facial vertical divergence, intermaxillary sagittal discrepancy, and maxillary and mandibular incisor positions. Landmarks, reference lines, and measurements used in this study are described in Table 2 and Figure 1 and Figure 2.

ANPg^ measures the angle between the Nasion–Point A line and the Nasion–Pogonion line. This angle was used to divide patients into three groups according to their skeletal sagittal class: Class III, with an ANPg^ equal to or less than −1°; Class I, with an ANPg^ between 0° and +4°; and Class II, with an ANPg^ equal to or greater than +5°. 

SN^GoGn measures the angle between the anterior cranial base (Sella–Nasion) and the mandibular plane (Gonion–Gnathion) and was used to classify the sample into three groups according to their facial divergence: hypodivergent, with an SN^GoGn equal to or less than +27°; normodivergent, with an SN^GoGn between +27° and +37°; and hyperdivergent, with a SN^GoGn equal to or greater than +37°. 

CoGoMe^ represents the angle between the Condylar axis (Condylion–Gonion) and the mandibular base (Gonion–Menton). Its reference value is 125.5°. The mandible of subjects with higher values tends to grow post-rotation (hyperdivergent growth pattern); on the contrary, subjects with lower scores have a hypodivergent growth pattern with an anterior mandibular rotation.

ANS-PNS^Ui and Go-Gn^Li angles were used to measure the inclination of the incisors’ long axis in relation to, respectively, the palatal plane and mandibular plane. ANS-PNS^Ui has a value of 110° ± 6°. Higher values indicate a proclined upper incisor; lower values suggest a retroclined maxillary incisor. The Go-Gn^Li angle indicates the proclination of the lower incisor, and 94° ± 7° represents the normal range of values for this angular measurement.

The position of the upper and lower incisors was measured as the UIP and LIP (Figure 2a,b).

**Figure 3:**
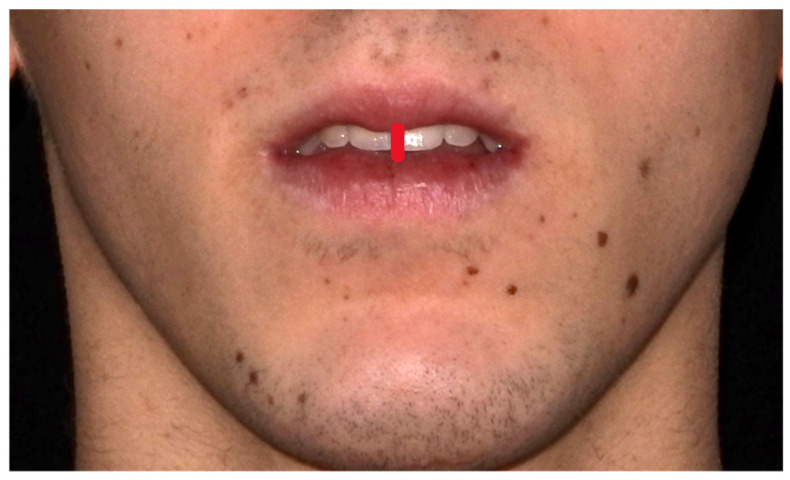
Lip incompetence was clinically evaluated based on a visual inspection of the patient’s inter-labial gap.

Clinical examination was used to assess the lip competence of the patients. Lip incompetence was clinically evaluated through visual inspection of the subject’s inter-labial gap, which is defined as the vertical distance between upper and lower lips at rest. A value of 0 mm identifies a lip competence, values from 1 to 3 mm identify a mild lip incompetence, and a value higher than 3 mm indicates a severe lip incompetence (Figure 3). The diagnosis of a lip incompetence was also recorded with extraoral pictures with the lips in a rest and usual position, and the authors of this study (RR and ACPM) assessed the extraoral pictures to evaluate lip incompetence.

### 2.4. Statistical Analysis

Dahlberg’s formula [30] and a paired Student’s *T*-test with the type I error set at 0.05 (*p* < 0.05) were used to assess the method of error. Hence, 101 randomly selected lateral cephalograms were reassessed by the same examiner after a memory washout period of at least 8 weeks.

Categorical variables were reported as frequencies and percentages, and continuous variables were reported as means and standard deviations if the data distribution was normal or as medians and interquartile ranges if the data showed a skewed distribution. The Shapiro–Wilk (SW) test was used to evaluate normality assumption.

One-way Analysis of Variance (ANOVA) and a Bonferroni test were used to check if the differences in LIP and UIP values between the examined groups were statistically significant. Differences in ANS-PNS^Ui and Go-Gn^Li between patients with hypodivergent, normodivergent, and hyperdivergent skeletal patterns were estimated using ANOVA and Fisher’s LSD procedure.

Linear regression analysis was performed to evaluate (1) how the UIP (used as a dependent variable) changed according to lip incompetence, ANPg^, SN^GoGn, and CoGoMe^; (2) how the LIP (used as a dependent variable) changed according to lip incompetence, ANPg^, SN^GoGn, and CoGoMe^; and (3) how the LIP and UIP changed according to Go-Gn^Li and ANS-PNS^Ui, respectively, in patients with different facial divergence. All the regression models were adjusted by gender and age.

The level of statistical significance was set at *p* < 0.05. Statistical analysis was performed using STATA version 14.0 (StataCorp LP, Stata Statistical Software, College Station, TX, USA).

## 3. Results

This study included 290 subjects (122 males (42.1%) and 168 females (57.9%)) aged 8 to 53 years (median 14; interquartile range 12–17).

The method error for the incisor’s position was between LIP 0.45 and UIP 0.52; the method error for the angular measurement was between 0.24 and 0.87.

There was no systematic error for any measurements (Student’s *t*-test, *p* < 0.05).

The mean values of the cephalometric measurements are summarized in Table 3. The sample was divided according to skeletal sagittal malocclusion, facial divergence, and lip competence criteria.

According to skeletal sagittal malocclusion, there were the following:

- 198 Class I subjects;
- 60 Class II subjects;
- 32 Class III subjects.

According to facial divergence, there were the following:

- 166 normodivergent subjects;
- 60 hypodivergent subjects;
- 64 hyperdivergent subjects.

According to lip competence, there were the following:

- 217 patients with competent lips;
- 48 patients with mild incompetence;
- 25 patients with severe incompetence.

Table 4 shows how LIP and UIP values varied according to lip incompetence, skeletal malocclusion, and lip incompetence.

Patients with severe lip incompetence showed the highest LIP and UIP values; particularly, it can be noted that the LIP increased with the worsening of lip incompetence. Class III patients had the lowest LIP value, while Class II patients and those with competent lips had the lowest UIP value.

A one-way ANOVA was performed to compare the effects of lip incompetence, skeletal sagittal malocclusion, and facial divergence on LIP and UIP values. The statistical analysis revealed that there was a statistically significant difference in the LIP (F (2, 287) = 7.8, *p* = 0.001) according to patients’ inter-labial gap, but no statistically significant difference was found for UIP values (*p* = 0.045). The Bonferroni test found that the mean value of the LIP was significantly different between patients with competent lips and those with severe incompetence (*p* < 0.001, 95% C.I. = −2.7, −0.6) and between patients with mild and those with severe lip incompetence (*p* = 0.011, 95% C.I. = −2.6, −0.2). There was no statistically significant difference in UIP values between patients with different skeletal malocclusions (*p* = 0.510) or between patients with different facial divergences (*p* = 0.870). However, subjects with different sagittal discrepancies (F (2, 287) = 21.8, *p* < 0.001) and patients with different vertical facial types (F (2, 287) = 16.9, *p* < 0.001) presented a statistically significant difference in LIP value. 

Simple linear regression was used to evaluate the influence of lip incompetence, SN^GoGn, ANPg^, and CoGoMe^ on LIP and UIP values. The results of the regression models are summarized in Table 5 and Table 6. The analysis showed that the LIP increased by 0.09 mm for each degree of SN^Go-Gn (*p* < 0.001), by 0.30 mm for each degree of ANPg^ (*p* < 0.001), and by 0.05 mm for every degree of CoGoMe^ (*p* < 0.001). In the matter of lip incompetence, it was found that the LIP increased by 0.67 mm and the UIP increased by 0.37 mm with each level of lip incompetence. On the other hand, no statistically significant relation was found between SN^GoGn, ANPg^, CoGoMe^, and UIP value.

Simple linear regression between the UIP and ANS-PNS^Ui found that the UIP increased by 0.04 mm for each degree of ANS-PNS^Ui. In analyzing this relation among groups of patients with different vertical skeletal patterns it was noted that the UIP increased by 0.08 mm for each degree of ANS-PNS^Ui in hypodivergent patients and 0.05 mm in those with normodivergent skeletal type. The relation between the UIP and ANS-PNS^Ui in hyperdivergent patients was not statistically significant (*p* = 0.838). The results are shown in Table 7.

Furthermore, it was found that the relation between LIP and GoGn^Li in hypodivergent, normodivergent and hyperdivergent patients was statistically significant. Data are presented in Table 8.

Lastly, Table 9 shows ANS-PNS^Ui and Go-Gn^Li values among patients with different facial divergences. The one-way ANOVA analysis revealed that there was a statistically significant difference in Go-Gn^Li (F (2, 287) = 21.6, *p* < 0.001) among patients with different SN^GoGn values. There was no statistically significant difference in ANS-PNS^Ui values among brachyfacial, normodivergent, and hyperdivergent patients (F (2, 286) = 1.18, *p* = 0.309).

## 4. Discussion

The aim of this study was to describe the distribution of the LIP and UIP in a sample of patients who underwent orthodontic treatment at the Orthodontics Department of the Federico II University Hospital in Naples. This study examined whether there were statistically significant differences between patients with various vertical and sagittal skeletal discrepancies and additionally assessed if these linear measurements varied in subjects with competent lips and those with mild and severe lip incompetence. It is interesting to note that the LIP and UIP do not describe the incisors’ inclination with respect to the palatal and mandibular plane but the position of these teeth relative to specific skeletal points of reference, such as the anterior nasal spine for the upper incisor and the Pogonion for the lower one. Since the LIP parameter considers only the position of the incisal margin relative to the Pogonion, it is not affected by the mandibular plane angle (Go-Me), thereby overcoming the primary limitation of the IMPA measurement, which is significantly influenced by the angle of the mandibular plane. The same applies to the UIP; by considering the distance between the incisal margin and Point A, the true position of the upper incisor can be determined without being concealed by the patient’s malocclusion. In a problem-oriented approach to orthodontic diagnosis, it turns out that a proper understanding of the nature of the patient’s malocclusion is essential for designing a treatment strategy based on the specific needs of the subject. Taking this into consideration, case studies and treatment planning should always be performed to recognize patients that can be successfully treated with orthodontic therapy alone and those who require orthognathic surgery. In many borderline cases, the discriminant factors could be just LIP and UIP values. According to Merrifield’s philosophy, an anterior limit of the dentition exists, and teeth should not be pushed forward off basal bone [31]. This concept is in line with numerous studies demonstrating that excessive proclination of the incisors, bringing the roots into contact with the bone cortex, may lead to iatrogenic sequelae, such as bone loss and root resorption [32,33]. Not taking into consideration the upper and lower incisor positions could also be dangerous for the patient’s aesthetic outcome. It is well known that the soft tissue contours of the face are determined not only by the skeletal foundation and soft tissue components (nose and chin, lip thickness, lip tonicity) but also by the dental support system provided by teeth; and for this reason, an extractive or a non-extractive treatment plan can have a considerable effect on facial aesthetics [34,35].

We analyzed a group of 290 patients and found out that patients with severe lip incompetence had the highest LIP and UIP values of the sample. Furthermore, the ANOVA test highlighted a statistically significant difference in LIP values among patients with lip competence, mild incompetence, and severe incompetence. Lip incompetence can result from several factors, including anterior open bite, excessive facial height, insufficient upper lip height, excessive overjet, and reduced functionality of the orbicularis oris muscle, which is a striated muscle that surrounds the mouth and is primarily responsible for lip movement. When this muscle’s function is compromised, it can contribute to the inability to fully close the lips.

Many of these factors are frequently observed in Class II Division 1 individuals. Leonardo et al. noted that incompetent lips are more prevalent in Class II patients with increased lower facial height and retrognathic Pogonion [36].

If untreated, lip incompetence can lead to complications, such as gingival inflammation related to incisors [24] and a higher risk of traumatic dental injuries [37]. The results of our study are in line with the findings of previous studies that analyzed the skeletal, dental, and facial soft tissue characteristics of patients with incompetent lips [26,38,39]. These studies showed that subjects with lip incompetence presented bimaxillary dentoalveolar protrusion of the upper incisors. Moreover, Drevensek et al. found that lower central incisors were more proclined in the incompetent lip seal group. Teeth are maintained in a state of equilibrium between the resting pressures of the lips, cheeks, and tongue, as well as the eruptive forces produced by the periodontal ligament [40,41]; hence, an imbalance in this system can alter their normal position. Over time, many authors have investigated the perioral muscular activity of subjects with lip competence and incompetence to find out if there were any differences between the two groups. Yamaguchi et al. found that subjects in the incompetent group presented an increased activity of the mentalis muscle at rest and a significantly greater proclination of the maxillary and mandibular incisors as compared to subjects in the control group [42]. This was in accordance with previous studies [43,44]. In future studies, it may be interesting to examine LIP and UIP positions in patients with tongue thrusting and atypical swallowing, which are other conditions that can alter the teeth’s state of equilibrium.

Regarding LIP values and different sagittal discrepancies, our study found that there was a statistically significant difference in the position of the lower incisor in skeletal Class I, Class II, and Class III malocclusions. According to the data, Class II patients showed the highest level of lower incisor protrusion, while Class III patients presented the lowest LIP value because of a retruded position of the incisor. Furthermore, it was found that each degree of increase in the ANPg^ resulted in an increase in the LIP by 0.3 mm. This confirms that the position of the mandibular incisors compensates for sagittal discrepancies in order to achieve stable occlusion and suggests that lower incisor orientation should be related to the surrounding structures of the patient rather than to normal values obtained from a heterogeneous group of subjects [45,46,47]. Class II malocclusion may result from different combinations of skeletal and dentoalveolar elements. Moyers et al. divided Class II patients into six horizontal types and five vertical types [48] to understand the real nature of the malocclusion and plan the most efficient therapy for each type. In a less specific way, Class II malocclusions reflect a maxilla–mandible skeletal discrepancy due to excessive maxillary growth and/or mandibular underdevelopment [49,50,51]. Class III patients usually present a flat or concave profile, reflecting the underlying skeletal condition: mandibular prognathism, maxillary deficiency, or a combination of both. In this study, we only investigated the relation between the upper and lower jaw, assessing the intermaxillary discrepancy for each subject (ANPg^) and not taking into consideration if the maxilla or the mandible was in a more advanced or retruded position in relation to the anterior cranial base (S-N). The results of our study may be explained through the dentoalveolar compensatory mechanism defined by Solow as a “system which attempts to maintain normal inter-arch relations under varying jaw relationships” [52]. Precisely, in this perspective, it stands to reason that in Class II malocclusions, where the maxilla is positioned anteriorly relatively to the mandible, lower incisors tend to protrude to achieve a proper occlusion, while in Class III, we found a lower LIP value, meaning that the mandibular incisor is in a more upright or retruded position with respect to Pogonion to balance the skeletal sagittal disharmony. Our findings are in accordance with numerous studies that showed the same incisors’ compensatory mechanism [21,53].

A successful treatment comes from a proper diagnosis, which is why clinicians need to analyze all the components of a patient’s malocclusion, not only sagittal discrepancies but also vertical problems. In this study, the facial divergence of subjects was measured through the SN^GoGn angle, and the sample was divided into three groups: hypodivergent, normodivergent, and hyperdivergent patients. A statistically significant difference in LIP values was found among the three groups; in particular, the LIP value increased by 0.09 mm for each degree of increase in the SN^GoGn angle. This means that hypodivergent subjects (SN^Go-Gn < 27°) will show a lower LIP value compared to normodivergent (SN^Go-Gn between 27° and 37°) and hyperdivergent (SN^Go-Gn > 37°) subjects. It is important to note that the LIP is strongly influenced by the Pogonion position and, consequently, the shape and width of the mandibular symphysis. Hyperdivergent patients show a narrow and elongated symphysis, which results in a less prominent chin. In contrast, in a hypodivergent facial pattern, the symphysis is wider and shorter [54]. The increased LIP value in hyperdivergent patients may be due to a more retrusive Pogonion’s position rather than a more proclined lower incisor. This is in accordance with the statistically significant difference in Go-Gn^Li among patients with higher, lower, and normal SN^GoGn angles that we found in our study. The hyperdivergent group showed the lowest Go-Gn^Li value, while the hypodivergent one presented the highest, meaning that an increase in facial divergence is related to the retroclination of the lower incisor. In this case, again, teeth compensate for the vertical discrepancy; lower incisors are in a more retroclined position to counter the possible development of an anterior open bite, which is a common finding in hyperdivergent patients [55]. These findings are in accordance with previous studies [56,57,58]. The LIP can also provide clinicians with a better understanding of the patient’s malocclusion, and it can be a support for the extraction non-extraction choice. In this study, we found that hyperdivergent patients presented lower Go-Gn^Li values and at the same time a more advanced lower incisor position relative to the mandibular symphysis. The presence of a small Go-Gn^Li could push the clinician toward a non-extractive treatment, while the LIP might be very advanced. Taking both parameters into consideration helps the clinician pinpoint the ideal position of the lower incisor, especially when the patient presents crowding in the lower arch. In this case, considering only the inclination of the lower incisor could lead the clinician to formulate a treatment plan centered around the proclination of the incisors to regain space, risking gingival recessions and loss of alveolar bone. In cases like this, considering that the LIP value can help us take note of the patient’s advanced lower incisor position relative to the symphysis, it allows us to carefully frame a treatment plan without altering the position of the incisors, therefore reducing the risk of iatrogenic damage.

Regarding UIP values in patients displaying different skeletal malocclusion and different facial divergences, no statistically significant variation was found according to the one-way ANOVA, possibly because of the greater compensating effect of the lower incisor on the vertical and sagittal disharmonies.

When it comes to the skeletal vertical discrepancy, it may be very interesting to find out the relation between upper and lower incisor positions and the facial growth pattern. The CoGoMe^ angle is a cephalometric parameter that allows the clinician to evaluate the mandibular morphology without the influence of other anatomical structures. A higher CoGoMe^ value indicates a short ramus height and a steeper mandibular plane [59,60]. It was found that the LIP presents a positive association with CoGoMe^, meaning that a mandibular growth pattern in backward rotation is related to a more advanced lower incisor. This angle can be used to predict the outcome of functional treatment in skeletal Class II malocclusions caused by a retrognathic mandible. The smaller the angle prior to treatment, the greater the improvement in the soft-tissue chin profile will be at the end [61]. According to Baccetti et al., there is a significant increase in the Pg-Nasion perpendicular and a relative forward movement in the Pogonion after functional therapy [62]; therefore, future studies should be conducted to determine if the LIP value also changes during this kind of treatment. 

The main limitation of this study was the less-than-varied ethnic background of its pool of subjects, which was entirely composed of southern Italians. Moreover, the presence of altering habits such as tongue thrusting, atypical swallowing, or thumb sucking was not taken into consideration and appropriately evaluated. These behaviors are known to influence the position of the incisors. Finally, considering the wide age range of the sample, we cannot generalize the data interpretation regarding the decision for extraction or non-extraction treatment. 

This study also presents many strong points. Due to its large sample size and wide age range, this study managed to accurately highlight the distribution of the UIP and LIP. By taking advantage of these new parameters, the clinician’s ability to analyze the position of the upper and lower incisors in relation to their underlying skeletal structure is greatly enhanced. These breakthroughs also open the way to more specialized and customized treatment plans, reducing the risk of iatrogenic damage and clinical error [63].

## 5. Conclusions

In conclusion, this study explored the distribution of UIP and LIP values across a sample of subjects who underwent orthodontic treatment at the Orthodontics Department of the University of Naples Federico II. The findings of this study reaffirm the established understanding of incisor positioning and inclination in relation to sagittal discrepancies and confirm how vertical discrepancies affect lip competence. Firstly, LIP values are increased among patients with different degrees of lip incompetence. Secondly, the statistically significant finding that the position of the lower incisors tended to be more protruded in Class II patients than in Class I patients and that it tended to be more retruded in Class III patients confirm the presence of the dentoalveolar compensation of skeletal sagittal discrepancy. Lastly, with respect to facial divergence, the LIP value was higher in hyperdivergent patients and showed a positive association with CoGoMe^. 

## Figures and Tables

**Table 1:** Age and gender distribution of the sample.

Variables	*N*	Mean ± SD	95% CI
**Age**	290	15.6 ± 6.0	14.9, 16.3
**Gender**		**Percentage %**	
Male	122	15.5 ± 5.2	14.5, 16.4
Female	168	15.7 ± 6.5	14.7, 16.6

Data are presented as mean ± standard deviation (SD) or percentage and 95% confidence interval (95% CI).

**Table 2:** Linear and angular cephalometric measurements used in this study.

Variable	Definitions
A-N-Pg (°)	Angle between the Nasion–Point A line and the Nasion–Pogonion line
S-N/Go-Gn (°)	Angle between the Sella–Nasion plane and the mandibular plane
Co-Go-Me (°)	Angle between the Condylar axis and the mandibular base
LIP (mm)	Distance between the perpendicular and the Frankfurt horizontal plane passing through the incisal margin of the mandibular central incisor and Pogonion
UIP (mm)	Distance between the perpendicular and the Frankfurt plane passing through the upper incisor’s margin and the anterior nasal spine
ANS-PNS/Ui (°)	Angle between the long axis of the maxillary central incisor and the palatal plane
Go-GN/Li (°)	Angle between the long axis of the mandibular central incisor and the mandibular plane

**Table 3:** Descriptive statistics of the assessed variables.

Variables	Mean (SD)	95% CI LL, UL
ANPg^ (°)	2.7 (3.2)	3, 2.3
SN^GoGn (°)	32 (6.9)	32.7, 31.2
CoGoMe^ (°)	127.2 (7.7)	126.3, 128.0
LIP (mm)	1.8 (2.1)	1.6, 2.0
UIP (mm)	1.0 (1.7)	0.8, 1.2
ANS-PNS^Ui (°)	111.7 (11.6)	110.3, 113.0
Go-Gn^Li (°)	95.2 (8.7)	94.2, 96.2

Data are presented as mean ± standard deviation (SD) and 95% confidence interval (95% CI) stating the lower limit (LL) and the upper limit (UL). ANPg^: the angle between NA and NPg. SN^GoGn: the angle between the SN plane and Go-Gn. CoGoMe^: the angle between CoGo and GoMe. LIP: lower incisor position (mm). UIP: upper incisor position (mm). ANS-PNS^Ui: the angle between the palatal plane and the long axis of the maxillary incisor. Go-Gn^Li: the angle between the mandibular plane and the long axis of the mandibular incisor.

**Table 4:** Distribution of the LIP and UIP according to lip incompetence, skeletal class, and facial divergence.

		LIP (mm)		UIP (mm)	
		Mean (SD)	CI 95% LL, UL	Mean (SD)	CI 95% LL, UL
**Lip Incompetence**	**Absence (*n* = 217)** **Inter-labial gap = 0 mm**	1.6 (2.0)	1.3, 1.8	0.8 (1.6)	0.6, 1.1
	**Mild (*n* = 48)** **Inter-labial gap = 1–3 mm**	1.8 (1.9)	1.2, 2.3	1.4 (1.7)	0.9, 1.9
	**Severe (*n* = 25)** **Inter-labial gap > 3 mm**	3.3 (2.0)	2.4, 4.1	1.5 (2.2)	0.5, 2.4
	***p*-Value**	**0.001**		0.045	
**Skeletal Class**	**Class I (*n* = 198)** **(0° < ANPg^ > +4°)**	1.7 (1.8)	1.4, 1.9	1.0, 1.7	0.8, 1.3
	**Class II (*n* = 60)** **(ANPg^ ≥ +5°)**	2.9 (2.3)	2.2, 3.5	0.8, 1.5	0.4, 1.2
	**Class III (*n* = 32)** **(ANPg^ ≤ −1°)**	0.1 (2.0)	−0.6, 0.8	1.2, 1.7	0.5, 1.8
	***p*-Value**	**<0.001**		0.510	
**Facial Divergence**	**Hypodivergent** **(*n* = 60)** **(SN^GoGn ≤ 27°)**	0.5 (2.1)	−0.05, 1.0	1.0, 1.8	0.5, 1.5
	**Normodivergent** **(*n* = 166)** **(27° < SN^GoGn > 37°)**	1.9 (1.9)	1.6, 2.2	1.0, 1.7	0.8, 1.3
	**Hyperdivergent** **(*n* = 64)** **(SN^GoGn ≥ 37°)**	2.4 (1.9)	1.9, 2.9	0.9, 1.6	0.5, 1.3
	***p*-Value**	**<0.001**		0.870	

Data are presented as mean ± standard deviation (SD) and 95% confidence interval (95% CI) stating the lower limit (LL) and the upper limit (UL). Differences in the LIP and UIP among individuals with different lip incompetence, skeletal class, and facial divergence were estimated using one-way ANOVA. Bold text indicates statistically significant differences.

**Table 5:** Results of the simple regression analysis for the different models analyzed.

				Age and Sex Adjusted		
Models	B	CI 95% LL, UL	*p*	B	CI 95% LL, UL	*p*
LIP/SN^GoGn	0.09	0.06, 0.12	**<0.001**	0.09	0.06, 0.12	**<0.001**
LIP/ANPg^	0.30	0.21, 0.34	**<0.001**	0.30	0.20, 0.34	**<0.001**
LIP/CoGoMe^	0.06	0.02, 0.09	**<0.001**	0.06	0.02, 0.09	**<0.001**
UIP/SN^GoGn	−0.01	−0.04, 0.02	0.513	−0.01	−0.04, 0.02	0.587
UIP/ANPg^	−0.06	−0.12, 0.01	0.076	−0.05	−0.12, 0.01	0.092
UIP/CoGoMe^	0.001	−0.02, 0.03	0.944	0.003	−0.02, 0,03	0.801

Data are presented as beta and 95% confidence interval (95% CI) stating the lower limit (LL) and the upper limit (UL). Bold text indicates statistically significant values (*p*-value < 0.05).

**Table 6:** Regression analysis of the LIP and UIP according to lip incompetence.

Models	B	CI 95% LL, UL	*p*
LIP/Lip incompetence	0.67	0.30, 1.04	**<0.001**
UIP/Lip incompetence	0.37	0.06, 0.69	**0.019**

Data are presented as beta and 95% confidence interval (95% CI) stating the lower limit (LL) and the upper limit (UL). Bold text indicates statistically significant values (*p*-value < 0.05).

**Table 7:** Results of UIP/ANS-PNS^Ui simple linear regression in patients with different facial divergences.

	Models	B	CI 95% LL, UL	*p*
	UIP/ANS-PNS^Ui (*n* = 290)	0.04	0.02, 0.06	**<0.001**
**Hypodivergent**	UIP/ANS-PNS^Ui (*n* = 60)	0.09	0.05, 0.13	**<0.001**
**Normodivergent**	UIP/ANS-PNS^Ui (*n* = 166)	0.05	0.03, 0.07	**<0.001**
**Hyperdivergent**	UIP/ANS-PNS^Ui (*n* = 64)	−0.003	−0.03, 0.03	0.838

Data are presented as beta and 95% confidence interval (95% CI). Bold text indicates statistically significant values (*p*-value < 0.05).

**Table 8:** Results of LIP/Go-Gn^Li regression in patients with different facial divergence.

	Models	B	CI 95% LL, UL	*p*
	LIP/Go-Gn^Li (*n* = 290)	0.09	0.07, 0.12	**<0.001**
**Hypodivergent**	LIP/Go-Gn^Li (*n* = 60)	0.15	0.09, 0.20	**<0.001**
**Normodivergent**	LIP/Go-Gn^Li (*n* = 166)	0.15	0.11, 0.18	**<0.001**
**Hyperdivergent**	LIP/Go-Gn^Li (*n* = 64)	0.01	0.05, 0.15	**<0.001**

Data are presented as beta and 95% confidence interval (95% CI) stating the lower limit (LL) and the upper limit (UL). Bold text indicates statistically significant values (*p*-value < 0.05).

**Table 9:** Distribution of Go-Gn^Li and ANS-PNS^Ui in patients with different facial divergences.

Variables		Go-Gn^Li		Ans-Pns^Ui	
		Mean (SD)	CI 95% LL, UL	Mean (SD)	CI 95% LL, UL
**Facial** **Divergence (SN^GoGn)**	**Hypodivergent** **(*n* = 60)** **(SN^GoGn ≤ 27°)**	98.9 (8.3)	96.7, 101.1	112.4 (10.4)	109.7, 115.1
	**Normodivergent** **(*n* = 166)** **(27° < SN^GoGn > 37°)**	96.0 (7.7)	94.8, 97.2	112.0 (11.1)	110.3, 113.7
	**Hyperdivergent** **(*n* = 64)** **(SN^GoGn ≥ 37°)**	89.5 (8.9)	87.3, 91.8	109.7 (13.7)	106.3, 113.2
	***p*-Value**	**<0.001**		0.309	

Data are presented as mean ± standard deviation (SD) and 95% confidence interval (95% CI) stating the lower limit (LL) and the upper limit (UL). Differences in the LIP and UIP among individuals with different lip incompetence, skeletal class, and facial divergence were estimated as appropriate using one-way ANOVA. Bold text indicates statistically significant differences.

## Data Availability

The data presented in this study are available upon request from the corresponding author.
